# Electroless-Deposited Platinum Antennas for Wireless Surface Acoustic Wave Sensors

**DOI:** 10.3390/ma12071002

**Published:** 2019-03-27

**Authors:** Erik Brachmann, Marietta Seifert, Niels Neumann, Nidal Alshwawreh, Margitta Uhlemann, Siegfried Bernhard Menzel, Jörg Acker, Steven Herold, Volker Hoffmann, Thomas Gemming

**Affiliations:** 1 Leibniz IFW Dresden, Helmholtzstraße 20, 01069 Dresden, Germany; e.brachmann@ifw-dresden.de (E.B.); m.uhlemann@ifw-dresden.de (M.U.); s.menzel@ifw-dresden.de (S.B.M.); v.hoffmann@ifw-dresden.de (V.H.); t.gemming@ifw-dresden.de (T.G.); 2 Chair of Radio Frequency and Photonics Engineering, TU Dresden, Helmholtzstraße 10, 01069 Dresden, Germany; niels.neumann@tu-dresden.de; 3 Industrial Engineering Department, German Jordanian University, Amman Madaba Street, P.O. Box 35247, Amman 11180, Jordan; nidal.alshwawreh@gju.edu.jo; 4 Faculty 2 Environment and Natural Sciences, Department of Physical Chemistry, Brandenburg University of Technology Cottbus-Senftenberg, Universitätsplatz 1, 01968 Senftenberg, Germany; joerg.acker@b-tu.de (J.A.); steven.herold@b-tu.de (S.H.)

**Keywords:** wireless SAW sensor, high-temperature, antenna, electroless deposition, platinum film

## Abstract

In an effort to develop a cost-efficient technology for wireless high-temperature surface acoustic wave sensors, this study presents an evaluation of a combined method that integrates physical vapor deposition with electroless deposition for the fabrication of platinum-based planar antennas. The proposed manufacturing process becomes attractive for narrow, thick, and sparse metallizations for antennas in the MHz to GHz frequency range. In detail, narrow platinum-based lines of a width down to 40 μm were electroless-deposited on γ-Al${}_{2}$O${}_{3}$ substrates using different seed layers. At first, the electrolyte chemistry was optimized to obtain the highest deposition rate. Films with various thickness were prepared and the electrical resistivity, microstructure, and chemical composition in the as-prepared state and after annealing at temperatures up to 1100 ${}^{\circ }$C were evaluated. Using these material parameters, the antenna was simulated with an electromagnetic full-wave simulation tool and then fabricated. The electrical parameters, including the S-parameters of the antenna, were measured. The agreement between the simulated and the realized antenna is then discussed.

## 1. Introduction

Due to their high accuracy, sensitivity, and ultra-fast response, surface acoustic wave (SAW) sensors are integrated in a wide range of advanced automotive, aerospace, telecommunication, and biotechnology applications [1,2]. Lab-on-chip devices based on SAW technology are currently implemented in a broad range of pressure, temperature, strain, chemical, and biological sensing systems [3,4,5,6,7,8,9]. Typically, SAW sensors are fabricated on piezoelectric substrates in advanced clean room facilities in a sequence of several lithography and coating steps. The feasibility of SAW technology in advanced bioengineering and temperature sensing applications can be further improved by optimizing the design to expand sensitivity and by controlling the internal structure to enhance thermal stability. Moreover, adopting simple fabrication processes and reducing material waste are essential to improve cost efficiency during mass production. Platinum is the material of choice for the fabrication of planar antenna components in wireless high-temperature SAW devices. Advantages of using platinum are that it has a high melting point and noble character, i.e., it is applicable under harsh environmental conditions as given in air or corrosive atmosphere and at high temperatures. Furthermore, platinum can easily be connected to platinum [10,11,12,13,14,15] or platinum alloy [5,16,17] based interdigital transducers in SAW devices by ultrasonic platinum wire bonding [18,19]. However, there are several design constraints such as a wide bandwidth requirement to cover any shift in the resonance frequency triggered by temperature variations. Also, it is important to have an excellent impedance matching between the antenna and the SAW sensor to obtain a high system performance. Up to now, many SAW sensors are manufactured and packaged without any antenna offering a 50 Ω interface where conventional (external, bulky) antennas may be connected [1,20] or are not intended for wireless read-out [2]. Attempts to lower the footprint of planar antennas include meander structures [21] and loop antennas [8]. However, to the best knowledge of the authors up to now not much effort has been spent on the antenna design in the past.

For the fabrication of these narrow and sparse antenna features, subtractive techniques are not attractive because of the substantial (circa 99%) material waste. On the other hand, additive processes such as electroplating or electroless deposition could be feasible alternatives. In both processes, a selective deposition of platinum can be obtained using a pre-structured seed layer. However, electroplating requires large area contacts with an external power source which are not often included within the antenna layout or may even deteriorate the performance characteristics. Moreover, maintaining a constant deposition rate to produce a homogeneous surface during the fabrication of long and narrow structures is not always possible. On the other hand, electroless deposition is an autocatalytic chemical process based on the instantaneously formed activity on the surface where the deposition takes place [22]. In comparison to electroplating, there is no need to supply an external current to start or maintain the deposition and electrons required to initiate the process are provided by a reducing chemical agent. However, there are some challenges that must be addressed regarding controlling deposition kinetics, bath stability, and the influence of additives on the homogeneity and electrical resistivity of the grown films.

In this work, we introduce the electroless deposition of platinum as a fabrication method for cost-effective SAW planar antenna components. The chemical bath used in this work was developed by Walter and Leaman [23] and later described in the electroless-plating handbook by Mallory [24]. A detailed investigation to optimize the process parameters and to describe the effect of annealing on the antenna characteristics is discussed. Moreover, a comparison of the electrical sheet resistivity of electroless-deposited and electrodeposited films is presented. Based on these measurements, a prototype of a platinum-based antenna produced by electroless deposition was simulated and then fabricated.

## 2. Materials and Methods

Most of the films investigated in this paper were deposited on γ-Al${}_{2}$O${}_{3}$-substrates (Rubalit 708S, CeramTec GmbH, Marktredwitz, Germany) with a purity of 96%, a total thickness of 0.6 mm, and with a maximum surface roughness of approximately 0.6 μm. Some other films were grown on a second type of γ-Al${}_{2}$O${}_{3}$-substrates with 99% purity (A479, Kyocera Corporation, Kyoto, Japan) with a smaller surface roughness of 0.06 μm, which were used to investigate the deposition kinetics. The pre-treatment steps for the substrates consisting of cleaning and thermal annealing were already described in former work [25]. Reference samples have been prepared on thermally oxidized Si substrates.

After pre-treatment, the substrates were structured using photolithography. For this purpose, the AZ5214E photoresist (Microchemicals GmbH, Ulm, Germany) was spin-coated on the substrate employing a POLOS SPIN150i SPIN COATER (Spincoating, Putte, The Netherlands), exposed by a maskless exposure unit (MLA 100, Heidelberg Instruments, Heidelberg, Germany), and then developed in AZ 726 MIF (Microchemicals GmbH, Ulm, Germany). All processes of structuring were carried out under clean room condition (ISO–5).

**Figure 1:**
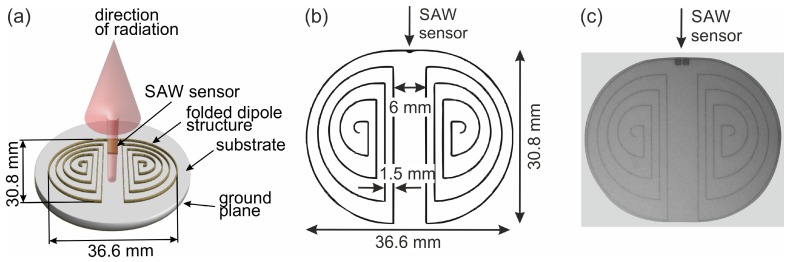
(**a**) Visualization of the principle of a folded dipole antenna structure on a substrate (not to scale). (**b**) Schematic drawing of the optimized antenna and (**c**) photo of a realized antenna structure.

Figure 1a presents a visualization of the principle of a folded dipole antenna structure on a substrate (not to scale). Figure 1b shows a schematic drawing of the optimized antenna design. An image of an antenna prepared with the electroless process is presented in Figure 1c. The intended line width is 50 μm. The antenna structure has been simulated in a 3D planar electromagnetic (EM) analysis software based on a Method of Moments (MoM) solver. Fed by a differential port, the matching of the antenna has been evaluated yielding information on the antenna resonance.

The coating of adhesion layers (Ti, W and Cr) and the Pt seed layer on the substrate was produced using magnetron sputtering and electron beam evaporation in a PVD cluster tool (CREAMET 350-CL 6, CREAVAC-Creative Vakuumbeschichtung GmbH, Dresden, Germany) with the same parameters as reported by Seifert et al. [25]. The 50 nm thick seed layer was structured using a standard lift-off process in a mixture of ethanol and acetone. Then, the required thickness of the Pt antenna structure was realized by electroless deposition using a slightly modified solution suggested by Walter and Leaman [23] and Rao et al. [22,26]. A stock solution was prepared by dissolving 2 g hexachloroplatinic acid (H${}_{2}$PtCl${}_{6}$ × 6 H${}_{2}$O) in 100 mL 4% HCl. In the electroless deposition experiments, a total of 25 mL solution volume containing 3.2 mL of the stock solution and 21.8 mL of distilled water was used. Before starting the deposition, the solution was heated in a thermalized water bath and the temperature was set between 30 and 90 ${}^{\circ }$C.

To determine the effect of additives on the deposition kinetics, the concentration of two additives was systematically varied. The concentration of the additive I (trisodium salt of 1,3,6-naphthalene-trisulfonic acid) was set to be between 0 and 1 g/L. The concentration of the additive II (sulfosalicylic acid) was varied between 0 and 1.75 g/L. The respective amount of the two additives was dissolved in the heated solution at the desired temperature. The substrate was then placed in the bottom of the heated beaker with the prepared solution. The last step involves adding 3.7 g/L hydrazine dihydrochloride to the heated solution. The deposition time was monitored to control the film thickness. After completion of the deposition, the sample was removed from the solution, washed with distilled water, and dried under a warm airflow.

After deposition, cross-sections of the film-substrate structure were prepared using the focused ion beam technique (FIB, Zeiss 1540 XB CrossBeam, Oberkochen, Germany) and imaged using scanning electron microscopy in the same device. Top-view images were acquired using a scanning electron microscope (SEM, Zeiss Ultra Plus, Oberkochen, Germany). The electrical resistance of the films was measured using the van der Pauw measurement method on planar and insulating substrates [27]. The measurements were performed by injecting a 5 or 10 mA direct current while reversing the current polarity at each measurement to suppress any constant thermoelectric offsets. The voltage was measured using a nanovoltmeter (2182A-Nanovoltmeter, KEITHLEY-TEKTRONIX Inc., Beaverton, OR, USA).

The film thickness of reference multilayers consisting of an adhesion and a Pt layer was determined using a mechanical profilometer (Dektak XT, Bruker Nano Surface Division) and X-ray fluorescence analysis (XRF, Fischerscope XUV 773, Helmut Fischer GmbH, Sindelfingen, Germany). Using the XRF spectra of the mechanically calibrated reference samples, the density of the materials, and the known architecture of the electroless-deposited samples their thickness was determined by XRF using the analysis software WinFTM V.6 SUPER. The film thickness of the prepared samples was measured by XRF approximately one hour after the deposition. The thickness scanning was done in steps of 0.5 and 0.6 mm in *x*- and *y*-direction, respectively. At each step, the measurement was repeated 5 times and the average thickness was determined.

The matching of the antenna (high frequency scattering parameter, *S* parameter |$S_{11}$|) was measured with vector network analyzers (E5071C ENA Vector Network Analyzer, Keysight Technologies, Santa Rosa, CA, USA and E8364B PNA Network Analyzer, Agilent Technologies, Santa Rosa, CA, USA) using high frequency probes (Air coplanar probe, Cascade Microtech, Beaverton, OR, USA) with a pitch of 0.75 mm for the measurement before tempering and standard RF cables soldered to the antenna pads for the measurement after tempering.

To detect organic residuals and contaminants in the deposited film, glow discharge optical emission spectrometry (GDOES) measurements were performed. Intensity-time profiles were captured with photomultipliers and a DC source at 10 mA and a pressure of 3 mbar with 2.5 mm diameter of the anode (GDA750, Spectruma Analytik GmbH, Hof, Germany). For elemental analysis, the following wavelengths were used: H 121.567 nm, Cl 133.572 nm, C 165.701 nm, S 180.731 nm, Pt 265.945 nm, Al 396.152 nm, Ar 415.859 nm, Cr 425.433 nm.

## 3. Results

### 3.1. Optimization of the Platinum Electrolyte and the Deposition Conditions

**Figure 2:**
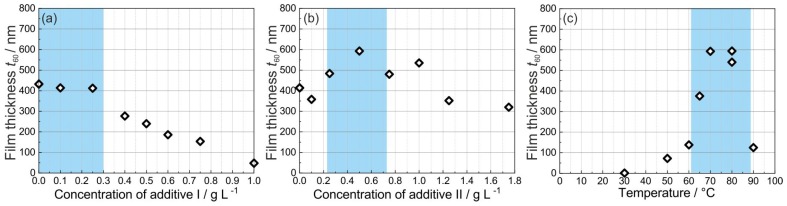
Film thickness $t_{60}$ after 60 min of deposition for various additive concentrations on thermally oxidized Si (100) substrates (100 nm W adhesion layer with 10 nm Pt seed layer): (**a**) variation of the additive I, the concentration of the additive II was zero, (**b**) variation of the additive II in the presence of the additive I (0.1 g/L). Both experiments were carried out at a bath temperature of 70 ${}^{\circ }$C. (**c**) Variation of the deposition temperature using a chemical bath containing additive I (0.1 g/L) and additive II (0.5 g/L). The colored background marks the parameter region which results in the highest film thickness.

To obtain a better understanding of the effect of the additives and the deposition temperature on the deposition rate, the concentration of the additives I and II as well as the bath temperature were varied. For these experiments thermally oxidized Si(100) substrates with a size of 10 mm by 10 mm covered with a 100 nm thick sputter deposited W adhesion layer and a 10 nm electron beam evaporated Pt seed layer were used. For the variation of the additives I and II, the temperature of the electrolyte during deposition was kept constant at 70 ${}^{\circ }$C. The results are summarized in Figure 2.

In the first step, the concentration of the additive I was varied between 0 and 1 g/L in the absence of the additive II. Figure 2a shows that the film thickness is about 420 nm when the concentration is between 0 and 0.3 g/L. As the concentration of the additive I increases, the film thickness decreases. Therefore, a concentration of 0.1 g/L of the additive I was chosen in the subsequent experiments. The concentration of the additive II was varied between 0 and 1.75 g/L. There is no linear dependence of the film thickness on the concentration (Figure 2b). An additive II concentration of 0.5 g/L appears to produce the largest film thickness. In a final step, the deposition temperature was varied between 30 and 90 ${}^{\circ }$C (Figure 2c). For these experiments, the optimized composition of the electrolyte contained 0.1 g/L of the additive I and 0.5 g/L of the additive II. The results reveal that a temperature between 70 and 80 ${}^{\circ }$C leads to the highest deposition rate. Based on these results, the additive concentrations were finally set to 0.1 g/L and 0.5 g/L for the additive I and the additive II, respectively. The deposition temperature was set to 70 ${}^{\circ }$C. Using these parameters, a film thickness of about 600 nm was attained on a 10 mm by 10 mm substrate after one hour of deposition.

It is also important to investigate the effect of the substrate type and roughness on the deposition kinetics. In this step, three different substrates were used: (i) single side polished single crystalline Si(100), (ii) γ-Al${}_{2}$O${}_{3}$ ceramic substrates with a purity of 96% Al${}_{2}$O${}_{3}$ and a surface roughness of $R_{a}=0.6$ μm, and (iii) γ-Al${}_{2}$O${}_{3}$ ceramic substrates with a purity of 99% Al${}_{2}$O${}_{3}$ and $R_{a}=0.06$ μm. The Si substrates were covered with 100 nm of sputtered W and 10 nm of Pt (e-beam evaporation). On both Al${}_{2}$O${}_{3}$ substrates, a 10 nm sputtered Cr adhesion layer followed by a 100 nm e-beam evaporated Pt seed layer was deposited.

**Figure 3:**
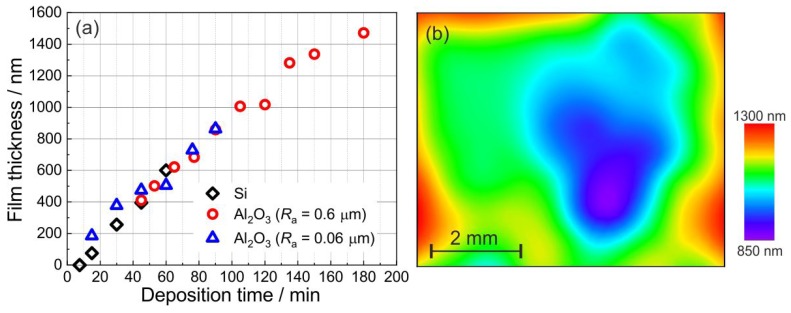
(**a**) Mean thickness of the electroless-deposited Pt-based film on a 100 nm Pt seed layer at an electrolyte temperature of 70 ${}^{\circ }$C in the presence of the additive I (0.1 g/L) and the additive II (0.5 g/L) and (**b**) film thickness distribution of an exemplary sample obtained from XRF measurements (deposition time 120 min, average thickness 1000 nm).

The deposition time was varied between 7.5 and 180 min. As shown in Figure 3a, no film growth was observed during the first 7.5 min of the deposition. During this so-called dead time chemical reactions at the substrate surface which are necessary to start the autocatalytic deposition of platinum are taking place. After this dead time nitrogen bubbles were observed at the substrate surface which indicate the start of the deposition process. Approximately, a constant film growth rate of about 11 nm/min during the first 60 min was achieved. For longer deposition times, the growth rate is slightly reduced (down to about 9 nm/min) which can be due to the decomposition of hydrazine which slows down the chemical reaction. Figure 3a shows that there is no significant influence of the substrate material or the roughness of the Al${}_{2}$O${}_{3}$ substrate on the deposition rate. However, it must be mentioned that for very thin films (in the order of the substrate roughness or lower) on rough Al${}_{2}$O${}_{3}$ substrates, an accurate determination of the film thickness is not possible. For this kind of substrates, only films with a deposition time of at least 50 min were considered.

Figure 3b demonstrates the thickness homogeneity of the sample deposited on a smooth Al${}_{2}$O${}_{3}$ substrate for 120 min with an average thickness of 1000 nm. It is obvious that the film is thicker at the edges of the sample. This finding is well known as the so-called dogbone effect. The heterogeneity of the structure is several orders of magnitude smaller than the wavelength of the antenna signal. Therefore, the propagating electromagnetic wave will not be influenced by the geometry but by the resulting change in bulk parameters such as conductivity. That has been taken into account in the simulations proving on the one hand the applicability of the process and on the other hand the potential of a process improvement (i.e., a smaller loss due to a better conductivity).

### 3.2. Electrical Resistance Measurements

**Figure 4:**
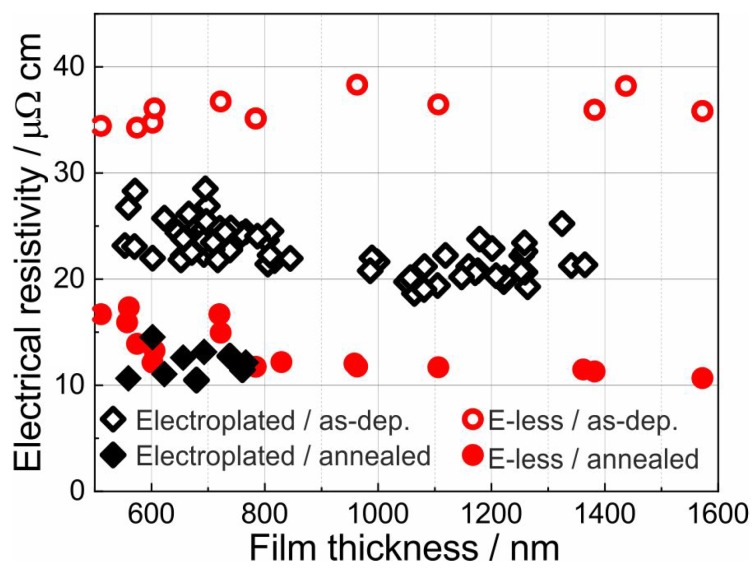
Electrical resistivity of electroless-deposited films in comparison with electroplated films for the as-prepared state and after annealing from room temperature up to 1000 ${}^{\circ }$C for 2 h.

The results of the electrical resistivity measurements of 500–1600 nm thick electroless Pt-based films are presented in Figure 4. For comparison, the electrical resistivity measurements of electroplated Pt films reported in our previous work are also included [25]. The resistivity was calculated using the measured average thickness and the electrical sheet resistance. In both deposition processes, the resistivity of the film in the as-deposited state was measured. In addition, the resistivity after 2 h heat treatment (in air, between 600 and 1000 ${}^{\circ }$C) was determined after cooling to room temperature. Former measurements of the resistivity of electroplated Pt films revealed that there is no significant difference in the resistivity of samples annealed at 600, 800 or 1000 ${}^{\circ }$C [25]. The results presented here show that the resistivity of the as-prepared electroless Pt-based films is in the range between 34 and 39 μΩcm. This value is about 10 μΩcm higher than those of electroplated films. Further analyses (see Section 3.3) reveal the presence of cracks in the electroless-deposited films. These cracks act as additional surfaces for electron scattering which increases the film resistivity. In both deposition processes, no significant changes in resistivity were observed with increasing the film thickness.

Annealing of metallic thin films after deposition normally leads to a reduction in electrical resistivity. Figure 4 clearly shows that by annealing, the resistivity of electroplated and electroless-deposited films has decreased to around 12 μΩcm. This value is close to the nominal resistivity of Pt (10 μΩcm) [22]. This indicates that a temperature treatment of the Pt-based antenna prior to its operation is essential to achieve the designed characteristics.

### 3.3. Film Morphology

**Figure 5:**
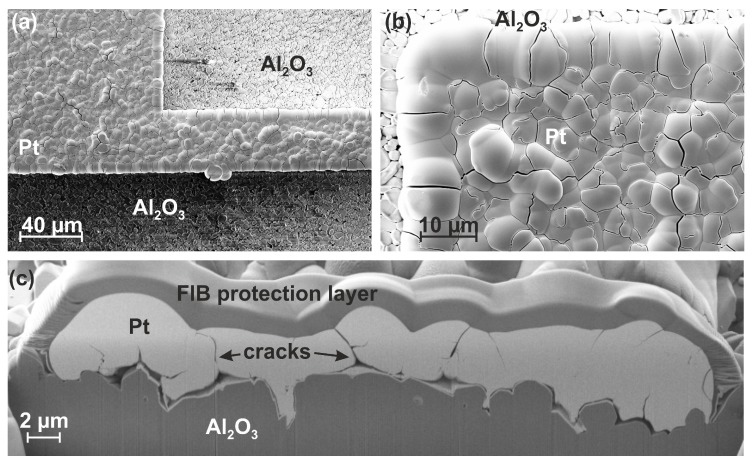
SEM micrographs (10 kV, SE2) of an electroless-deposited Pt-based antenna structure on an Al${}_{2}$O${}_{3}$ ($R_{a}=0.6$ μm) substrate, (**a**,**b**) top-view images, (**c**) SEM (3 kV, SE2) image of the cross section.

As an example of the resulting film morphology, top-view as well as cross-section images of an electroless-deposited film are depicted in Figure 5. The films were grown on a structured film system consisting of a 25 nm Ti adhesion and a 50 nm Pt seed layer on an Al${}_{2}$O${}_{3}$ ($R_{a}=0.6$ μm) substrate. The deposition time of the electroless-deposited film was 60 min. The overview image in Figure 5a shows a rough structure and the presence of cracks within the metallization. The image with higher magnification in Figure 5b reveals the presence of structures with a diameter of up to several μm which are separated by cracks. Furthermore, there is a fine-grained sub-structure with grain sizes of a few tens of nm. In the cross-section image in Figure 5c, cracks are visible which, in some cases, extend through the whole film thickness. Some smaller cracks appear at the upper or the lower parts of the film. The origin of these cracks are most likely gas bubbles which are generated by chemical reactions. Hydrazine acts as a reducing agent and provides the electrons for the reduction of the Pt-complexed ions to form metallic Pt. During the decomposition of the hydrazine nitrogen is formed. Another mechanism is based on the additional formation of atomic hydrogen during the oxidation process [28,29]. Atomic hydrogen diffuses in the Pt layer or recombines to molecular hydrogen generating gas bubbles. In particular, the atomic hydrogen leads to internal stress, crack formation, and embrittlement. During the heat treatment hydrogen diffuses out and the cracks disappear by surface diffusion processes accompanied by a remarkable grain growth and the formation of globular pores. A decrease of the hydrogen content in the deposited layer after the heat treatment was proven by GDOES, as described in Section 3.4. In agreement with the XRF thickness scans, Figure 5c also demonstrates the significant difference in film thickness at the edges compared to that in the center.

The initial experiments for the determination of the deposition rate on 10 mm by 10 mm substrates after 60 min of deposition resulted in an average film thickness of about 600 nm. For the structured sample shown in Figure 5 the film thickness is about 2 to 4 μm which is much higher than the expected value. A reason for the strongly increased deposition rate for structured samples are much more short diffusion paths for the supply of Pt to the growing regions if only narrow lines are deposited instead of an extended area.

For the antenna presented in Figure 5 the seed layer was structured as a curved line with a width of 30 μm (antenna for 433 MHz frequency band). Due to the isotropic growth of the electroless plated films and an increased growth rate at the edges of the antenna line its width increases to about 40 μm at the end of the deposition. This broadening effect must be taken into account while designing the antenna.

**Figure 6:**
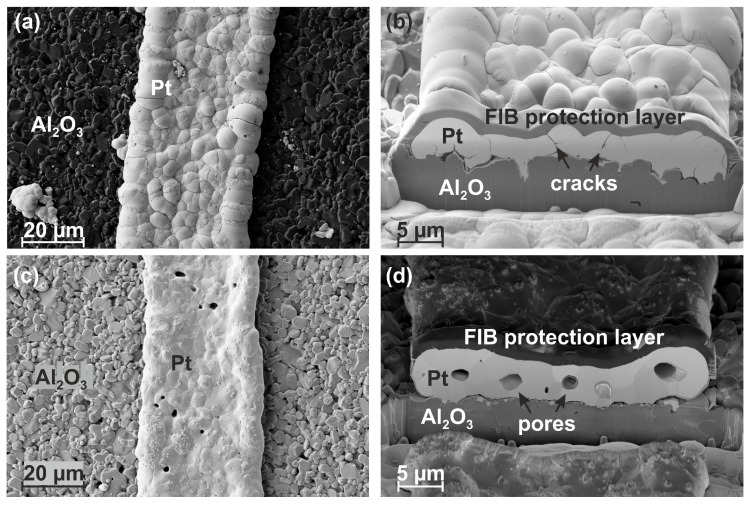
Surface and cross-section images (3 kV, SE2) of electroless-deposited antenna structures: (**a**) and (**b**) in the as-prepared state, (**c**) and (**d**) after annealing for 2 h at 1000 ${}^{\circ }$C in air.

Figure 6 presents a comparison between the electroless-deposited antennas in the as-prepared state (Figure 6a,b) and after annealing at 1000 ${}^{\circ }$C for 2 h (Figure 6c,d). After annealing, the microcracks have largely disappeared and pores have formed especially at triple points. The transformation from long gaps to globular pores was likewise observed for Pt wedges on Pt thick films [19]. The grain size is in the order of a few μm. Because of this strong grain growth and the reduced density of microcracks, the electrical resistance of these films decreases as observed in the van der Pauw measurement results (see Figure 4). The pores are much smaller (typically < 10%) than the width of the Pt waveguide and their dimension is insignificant compared to the free-space wavelength of the operation frequency (433 MHz leading to a wavelength of 69 cm). Therefore, no extra effect on the antenna characteristics due to the pores is expected [30].

### 3.4. Chemical Composition Analysis

In electroless and electroplated Pt films, organic components in the electrolyte will be embedded in the deposited films. These residuals can significantly influence the electrical resistivity and the thermo-mechanical properties of the film. To study this issue, the composition of as-prepared and annealed platinum-based films was measured using GDOES. This system allows an accurate detection of most elements in the periodic table. For this purpose, the setup was set to analyze an area of about 2.5 mm in diameter by depth profiling.

**Figure 7:**
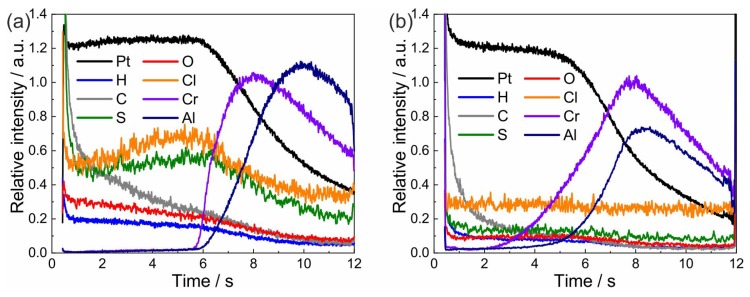
GDOES depth profiles of an electroless-deposited sample (**a**) in as-deposited state and (**b**) after annealing at 1100 ${}^{\circ }$C for 4 h in air.

The depth profiles which resulted from analyzing an as-prepared sample as well as a heat-treated sample (4 h at 1100 ${}^{\circ }$C in air) are presented in Figure 7. For these samples, a Cr adhesion layer on top of the Al${}_{2}$O${}_{3}$ substrate was used. When evaluating these measurements, one must take into account that there is no direct relation between the intensity and the concentration of the element in the sample. Quantification needs a calibration of the GDOES instrument with samples of a defined composition and sputtering rate. However, qualitative measurements allow the drawing of conclusions on the depth distribution of the elements and to compare different samples. Hereby, the ratio of intensities is proportional to the ratio of concentrations.

In the as-prepared samples (see Figure 7a), the presence of H, O, Cl and S is proven in the Pt-based layer. In addition, C is detected with a higher concentration at the sample surface. However, a continuous decrease in the signal intensity during the sputtering process was observed. As expected from the sample architecture, Cr is detected before Al. GDOES measurements are not sensitive to the presence of O in Al${}_{2}$O${}_{3}$, which explains why there is no increase of the O signal when the substrate is reached.

After the heat treatment (Figure 7b), the intensity of H, O, Cl, and S is clearly reduced compared to the as-prepared state. Moreover, the intensity of the C signal shows a faster decay. These results demonstrate that the heat treatment leads to a significant reduction in the concentration of organic residuals in the Pt-based metallization.

### 3.5. Antenna Simulation and Fabrication

In the final step, the results obtained from the discussed experimental investigations (the optimized electrolyte composition and deposition temperature) are used to manufacture a prototype of an antenna for wireless sensing applications.

SAW sensors are wirelessly powered by external readers via a radio signal. This signal is received by an antenna, transferred to the sensor, processed there, transferred back to the antenna, and then transmitted. Therefore, the antenna impedance must be matched to the sensor to avoid reflections. As reflections lower the available power and, consequently, the signal-to-noise ratio of the sensor response, an antenna matching of better than −10 dB is a usual design goal.

**Figure 8:**
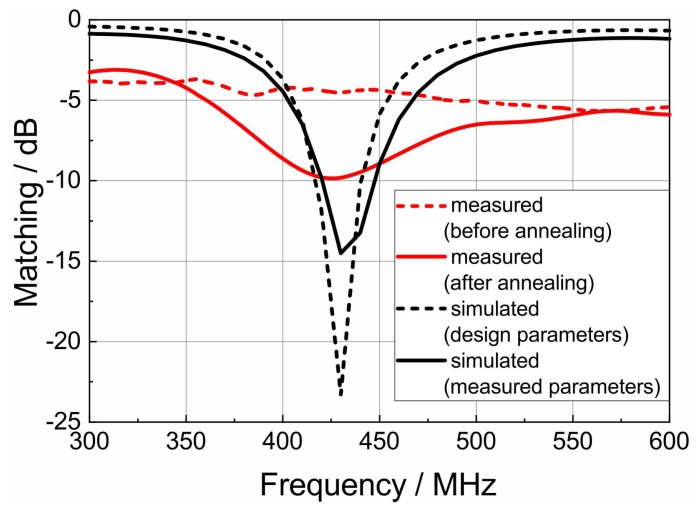
Matching of antenna ($S_{11}$ parameter). Presented are measurements before and after tempering as well as a simulation with parameters used for the design and a simulation with the adjusted parameters according to the achieved values in the manufacturing.

For the specified application, the following requirements for the antenna must be fulfilled: First, the design should be optimized for small dimensions. This lowers the cost, maximizes the yield on the substrate and enables a compact setup of the SAW device as presented in the schematic drawing shown in Figure 1b or as a photo of a realized antenna in Figure 1c. Second, the feeding should be differential as provided from the SAW sensor with 50 Ω impedance. Third, to shield the antenna from being affected by any metal structures on its backside and to improve the radiation characteristic to the frontside, a metal layer at the backside should be deposited. This metallization of the backside consists of a 25 nm Ti adhesion layer followed by 175 nm thick Pt layer deposited by electron beam evaporation. Taking into account these requirements, a folded dipole structure with a backside metallization is used. A standard $\lambda_{l}$/2-dipole for the operating frequency $f_{r}=430$ MHz on a substrate with the permittivity $\epsilon_{r}=9.8$ (γ-Al${}_{2}$O${}_{3}$) would have a length of

(1) $$\frac{\lambda_{l}}{2}=\frac{c_{0}}{2f_{r}\sqrt{\epsilon_{r}}}\approx 22\;\mathrm{cm},$$

where $c_{0}$ is the speed of light in vacuum. Folding that dipole into a circle with a diameter of less than 4 cm is possible as shown in Figure 1b. The advantage of a circular shaped antenna device is that this shape allows a positioning e.g., in smooth drilled holes. The simulation of the antenna matching (shown in Figure 8, black dotted line) has been carried out using conventional material parameters ($\epsilon_{r}=9.8$, tanδ=0.001 for the substrate, 10 μΩcm conductivity for the lines) leading to the structure shown in Figure 1b with a line width of 50 μm.

Measurements using a vector network analyzer have been carried out before and after heat treatment at 1100 ${}^{\circ }$C for 2 h in ambient atmosphere. Figure 8 shows that improving the conductivity enables the realization of an antenna resonance with sufficient matching (around −10 dB in the desired ISM band around 433 MHz). The measured antenna matching agrees well with the predicted matching from the simulation results. Analyzing the manufactured antenna structure showed that the line width was 40 μm instead of 50 μm. Furthermore, the conductivity was 12 μΩcm instead of 10 μΩcm used for the antenna design. The substrate permittivity remained unchanged ($\epsilon_{r}=9.8$, tanδ=0.001). The changed geometry and material parameters affected the matching and led to an increased loss (Figure 8, black line). Thickness variations were not taken into account in the simulation but are expected to broaden the resonance and decrease the matching only slightly. In the measurements, higher loss resulting in a broader resonance and a worse matching can be seen (Figure 8, red line). This could be related to the transition between the vector network analyzer and the antenna which adds additional loss and reflections which could not be fully de-embedded. Deembedding the interface between the probes and the manufactured structure on the substrate would imply to realize calibration structures with the same process on the same structures. Commercial calibration substrates with different substrate materials and metals cannot be used here because of their inherently different field distribution and therefore different realized impedance. In a process without vias, it is difficult to manufacture short and open calibration standards. Therefore, only the multiline Thru-Reflect-Line (TRL) calibration standard is an option [31,32]. However, these calibration structures consume much space and for this reason, the authors decided to work without a calibration at the cost of a slightly reduced measurement accuracy. Thus, it is expected that the performance with a connected SAW sensor device is better than the measurements presented here. Nevertheless, the measured matching of about −10 dB is sufficient for many applications.

## 4. Conclusions

Electroless Pt-based films with thicknesses from several 100 nm up to 1.6 μm have been deposited on various types of substrates (Si, Al${}_{2}$O${}_{3}$ ceramics with a different roughness and adhesion layers). A composition of the electrolyte based on hexachloroplatinic acid with hydrazine as reduction reagent containing 0.1 g/L of the additive I (trisodium salt of the 1,3,6-naphtalene-trisulfonic acid) and 0.5 g/L of the additive II (sulfosalicylic acid) and a deposition temperature of 70 ${}^{\circ }$C resulted in the highest deposition rate of Pt. A heat treatment at up to 1100 ${}^{\circ }$C for 2 h demonstrated a high-temperature film stability. The annealing also caused a reduced electrical resistivity which is close to the nominal resistivity of Pt (10 μΩcm). During heat treatment, cracks which are formed in the film structure during the deposition process convert into pores which, however, are not expected to influence the antenna characteristics. The high-temperature treatment stabilizes the microstructure of the layers for high-temperature applications. The results show that we successfully prepared a Pt-based antenna by structuring a thin PVD seed layer and subsequently applying electroless deposition to increase the antenna thickness. This planar antenna design with a backside metallization for the resonance frequency of 433 MHz was realized. An antenna matching of about −10 dB was measured in a reasonable agreement with the simulated structure based on the design parameters.

## Abbreviations

The following abbreviations are used in this manuscript:

FIB	focused ion beam
GDOES	glow discharge optical emission spectrometry
PVD	physical vapor deposition
SAW	surface acoustic wave
SEM	scanning electron microscope
XRF	X-ray fluorescence analysis
